# Cis-regulatory elements in CAMTA-mediated stress signalling: mechanisms and prospects for CRISPR-based crop improvement

**DOI:** 10.3389/fpls.2026.1864496

**Published:** 2026-06-10

**Authors:** Hena Gain, Joydeep Banerjee

**Affiliations:** Agricultural and Food Engineering Department, Indian Institute of Technology Kharagpur, Kharagpur, India

**Keywords:** calcium signalling, CAMTA, CGCG motif, cis-regulatory elements, CRiSPR/Cas, crop engineering, genome editing, stress tolerance

## Abstract

Enhancements in crop resilience strategies that maintain production are essential to address the challenges posed by climate change and increasing food consumption. Calcium-dependent signaling networks are essential for plant responses to abiotic and biotic stressors, with calmodulin-binding transcription activator (CAMTA) transcription factors serving as crucial regulators within this framework, as these factors govern gene expression through specific cis-regulatory elements located in promoter regions. Recent investigations have expanded to include CAMTA-binding motifs as the stress-responsive cis-regulatory modules across several plant species under examination. These findings indicate that CAMTA-associated cis-elements, comprising CGCG motifs and ABA-responsive regions, facilitate the integration of environmental signals that influence transcription. Cis-regulatory elements (CREs), such as promoters, enhancers, silencers, and insulators, control the exact timing and location of stress-responsive gene expression in plants. Recent breakthroughs in genome editing have enabled the direct manipulation of these cis-regulatory areas, facilitating precise control over gene expression. This work presents an overview of CAMTA structures, their interaction with promoter cis-regulatory regions, and the potential for promoter cis-element engineering to enhance agricultural performance under diverse settings. It emphasizes CRISPR-based strategies for precise CRE modifications and highlights the role of CAMTA in identifying stress-responsive regions. This establishes the foundation for the advancement of next-generation stress-resilient crops, which will ensure food security.

## 1 Introduction

Plants encounter numerous environmental challenges, including drought, salinity, heat, cold, and diseases, which impede their productivity and jeopardize global food security (Miller et al., 2010). It is essential to devise innovative crop modification strategies to enhance crop resilience to stress while maintaining their agronomic performance amid increasing climate variability (Reynolds et al., 2012). A critical facet for the crop modification to tackle stress is transcriptional regulation, particularly the importance of cis-regulatory elements (CREs), which regulate the timing and intensity of gene expression (Cui et al., 2023). Molecular switches, including promoters and enhancers, participate in this process. These switches guarantee the integration of environmental signals into plant responses (Debnath et al., 2024).

Current agricultural techniques, especially conventional breeding procedures, are becoming more inadequate for rapidly adapting to changing climates (Ahmar et al., 2020). Consequently, advanced biotechnological methods, specifically genome editing applications focused on CREs, are emerging as transformational strategies. Non-coding DNA sequences termed CREs, encompassing core promoter elements, enhancers, and silencers, serve as the molecular determinants for gene control. Variations in CREs account for a substantial proportion of the phenotypic diversity utilized in crop domestication (Xu and Liu, 2024). The precise engineering of CREs gives a promising prospect for agricultural enhancement.

CAMTA proteins, known as calmodulin-binding transcription activators, perform a pivotal role as transcriptional regulators involved in stress responses (Du et al., 2009). These regulators respond to calcium signals triggered by external stimuli, thereby linking signal perception to gene expression regulation. CAMTA proteins facilitate combinatorial regulation by binding to certain promoter motifs in conjunction with other regulatory elements (Yu et al., 2023; Cai et al., 2024). The optimal approach to comprehend their regulatory scope is as a multifaceted system of cis regulation, centered on the identification and manipulation of CREs associated with stress responsiveness.

CAMTAs engage with stress-responsive CREs by identifying certain motifs, including the CGCG core and the ABA-responsive element (ABRE), in a calcium-dependent fashion to modulate stress-response pathways (Abdel-Hameed et al., 2024). This review aims to examine the structural recognition of CREs by CAMTA proteins, the functional significance of these interactions in various crop species facing diverse stressors, and the utilization of CRISPR/Cas technology and synthetic biology for the design of these elements. Furthermore, it also addresses the issues related to biosafety, public acceptance, and regulatory ramifications associated with CRE-modified crops. The ultimate aim is to translate the insights acquired from CAMTA-CRE interactions into successful strategies for cultivating resilient crops that align with global sustainability objectives.

This review analyzes CAMTA proteins from a cis-regulatory viewpoint. It also elucidates the domain structure, DNA-binding specificity, promoter topologies, and expression patterns unique to particular species. Furthermore, the investigation on genome editing and the engineering of synthetic promoters was included, illustrating the mechanism of the alteration for CAMTA-associated CREs, with the aim of improving plant resilience to fulfil the growing demands of the global population.

## 2 CIS-regulatory elements: types, architecture, and relevance to stress gene expression

### 2.1 Classes of CREs in plant stress-responsive promoters

Plant promoters that control stress-responsive gene expression are modular assemblies of distinct CREs that work together to determine the specificity, amplitude, and timing of transcriptional output (Nayak et al., 2025). A thorough study of the variety and combinatorial logic of stress-responsive CREs across abiotic and biotic conditions highlights that promoter specificity is determined by element identity, copy number, spatial arrangement, and interactions with adjacent sequences (Ding et al., 2026). The main types that are important to CAMTA-mediated regulation are:

#### 2.1.1 Core promoter elements

TATA boxes and initiator (Inr) sequences are core promoter elements that set the transcription start site (TSS) and the construction of the basal transcriptional machinery (Szymczyk and Majewska, 2024). Modifications to the core promoter should change the amount of expression without changing the tissue or stress specificity.

#### 2.1.2 ABA-responsive elements

The canonical ABRE (CACGTG, a G-box variation) and its coupling element ABRE-CE [(C/A)ACGCG(T/G/C)] are among the most well-known stress-responsive CREs in plants. These are responsible for ABA-dependent signaling of drought, salinity, and cold (Gómez-Porras et al., 2007). CAMTAs bind directly to ABRE-CE sequences, and these elements respond to Ca^2+^ (Finkler et al., 2007; Wankerl, 2016).

#### 2.1.3 CGCG-core elements

The CGCG motif and its longer versions [CG(C/T)G, (G/A/C)CGCG(T/G/C)] are the major places where the CAMTA CG-1 domain binds (Yang and Poovaiah, 2002; Doherty et al., 2009). The promoters of cold-responsive CBF genes, defense-related EDS1, and other stress regulons all have these parts (Rahman et al., 2016a). These are the primary CRE targets for CAMTA-based regulatory engineering.

#### 2.1.4 Ca^2+^-responsive elements

These are different from ABREs since they only respond to Ca^2+^ signatures. Evidence also shows that CGCG and ABRE-CE elements are also CaREs (Kaplan et al., 2006; Finkler et al., 2007). This connects CAMTA-mediated transcription to the larger Ca^2+^ signaling network (Wankerl, 2016).

#### 2.1.5 W-box (TTGAC/T)

WRKY TFs broadly recognize W-boxes, which are often found with CGCG elements in the promoters of defense genes (Park et al., 2005). This facilitates biotic stress responses to be controlled in several different ways.

#### 2.1.6 DRE/CRT elements (GCCGAC)

Dehydration-responsive elements (DREs) interact with CBF/DREB transcription factors (TFs). Their promoter design commonly overlaps with CAMTA-responsive modules, notably in cold signaling (Doherty et al., 2009).

### 2.2 Promoter architecture and CRE combinatorics

Stress-responsive plant promoters function not through isolated single CREs but through combinatorial CRE modules (Zou et al., 2011). The *CBF1* (C-repeat binding factor 1) promoter exemplifies this concept, and it consists of overlapping CGCG-core elements recognized by AtCAMTA3, in addition to ICEr elements for ICE1 binding, enabling the integration of cold-sensing and Ca^2+^ signals into a cohesive cold-acclimation transcriptional response (Doherty et al., 2009). Drought-responsive promoters generally contain tandem ABRE elements, each requiring a coupling element for optimal operation, so creating cooperative CRE clusters that improve signal-to-noise ratios under conditions of water scarcity (Yang et al., 2026).

Comprehending this CRE combinatorics is crucial for engineering, as altering a single CGCG motif usually influence the reliance of a gene on Ca^2+^/CAMTA without inhibiting its basal expression. In contrast, changing a connected ABRE-ABRE-CE module should change the thresholds for ABA sensitivity by a lot. This modularity is what makes CRE engineering easy to work with and change, unlike changing the coding sequence. **Table 1** provides a concise overview of the major classes of CRE, consensus sequences, binding TFs, and engineering potential that are important for CAMTA-mediated stress regulation.

**Table 1:** Essential cis-regulatory components linked to the expression of stress-responsive genes in plants as controlled by CAMTA.

CRE type	Consensus sequence	Primary TF	Stress relevance	Engineering potential
CGCG-core	(G/A/C)CGCG(T/G/C)	CAMTA (CG-1 domain)	Cold, drought, biotic stress	High because direct editing changes CAMTA affinity
CG(C/T)G extended	CG(C/T)G	AtCAMTA1, OsCBT	Cold, ABA signalling	High because synthetic promoter design target
ABRE	CACGTG (G-box)	bZIP (ABI5)	ABA, drought, salinity	High because ABRE copy-number editing alters ABA threshold
ABRE-CE	(C/A)ACGCG(T/G/C)	CAMTA with bZIP	ABA/Ca^2+^ convergence	Very High because dual TF binding, combinatorial target
DRE/CRT	GCCGAC	CBF/DREB	Cold, drought	Moderate because editing alters CBF responsiveness
W-box	TTGAC/T	WRKY	Pathogen defense	Moderate because defense pathway tuning
CaRE	CGCG/ABRE-CE	CAMTA	Ca^2+^-specific responses	High because Ca^2+^-signal decoupling/enhancement

## 3 CAMTA domain architecture and cre recognition: structural basis for engineering

### 3.1 The CG-1 domain: primary interface with cis-regulatory DNA

The defining feature of CAMTA transcription factors is the CG-1 domain, a 130 amino acid N-terminal module that facilitates direct, sequence-specific binding to CGCG-core cis-regulatory elements (Yang and Poovaiah, 2002). The CG-1 domain possesses a bipartite nuclear localization signal (NLS) and establishes sequence-specific interactions with the CGCG motif, influenced by the adjacent nucleotide context, elucidating the variation in CRE preferences among different CAMTAs (Finkler et al., 2007; Liu et al., 2025). The CG1 domain is the defining characteristic of the CAMTA family and its presence is both essential and adequate for classifying a protein as a CAMTA. Also, the CRE-binding specificity of CG1 serves as the molecular basis for CRE-targeted engineering.

From a CRE engineering perspective, the binding preferences of the CG-1 domain specify the promoter elements that should be altered to affect CAMTA-dependent gene regulation. Changes in the CGCG core, like changing CGCG to CTCG, stop CG-1 from binding, which makes the downstream gene CAMTA-independent (Liu et al., 2025). On the other hand, adding synthetic CGCG arrays to minimal promoters normally make constitutive or inactive promoters respond to Ca^2+^/CAMTA, as shown by *in vitro* binding tests with real plant gene promoters (Doherty et al., 2009).

AtCAMTA3 (also known as AtSR1) binds to the consensus sequence (G/A/C)CGCG(T/G/C) in *Arabidopsis* (Fang et al., 2022). AtCAMTA1 and rice OsCBT, on the other hand, detect the expanded core CG(C/T)G (Choi et al., 2005; Finkler et al., 2007). In tea (*Camellia sinensis*), CAMTA binds to a G-box (CACGTC) cis-element in the promoter of the Fluoride Export Gene 1 (*FEX1*) to inhibit its transcription, while Calmodulin 1 (CAM1) physically interacts with CAMTA to create a repressor complex. This CAM1–CAMTA–FEX1 module controls intracellular fluoride homeostasis via Ca^2+^-mediated CRE regulation (Li et al., 2025). The subtle differences in CRE identification between CAMTA paralogs lay the groundwork for paralogue-specific promoter engineering. By designing promoters with tailored CGCG variants, it is possible to selectively recruit in specific CAMTA family members that show distinct Ca^2+^-response kinetics.

### 3.2 TIG domain: cooperative DNA binding and promoter architecture implications

The transcription factor immunoglobulin (TIG) domain, positioned next to the CG-1 domain, facilitates non-specific DNA binding and protein dimerization (Müller et al., 1995). The CG-1 and TIG domains together form a composite DNA-binding module, with TIG enhancing DNA affinity, thereby enabling the CG-1 domain to specifically recognize the CGCG sequence (Finkler et al., 2007; Gain et al., 2022). The cooperative nature of CG-1/TIG binding indicates that CAMTA binding to CREs is affected by the local promoter architecture along with the distance between CGCG elements, neighbouring sequences, and DNA topology all influence binding efficiency.

This has explicit implications for synthetic CRE design: optimal spacing between tandem CGCG motifs and between CGCG and ABRE-CE elements must be preserved or deliberately created to augment TIG-assisted cooperative binding. About 25% of plant CAMTAs lack the TIG domain, indicating that these paralogs have evolved for CREs in distinct chromatin contexts (Pant et al., 2018; Xiao et al., 2021).

### 3.3 IQ motifs and CaMBD: Ca^2+^/CaM-dependent activation and its CRE implications

CAMTA proteins have one or more C-terminal IQ motifs (IQXXXRGXXXR) and a calmodulin-binding domain (CaMBD) that is 22 amino acids long. These components enable both Ca^2+^-independent and Ca^2+^-dependent binding of calmodulin (CaM) (Bähler and Rhoads, 2002; Choi et al., 2005). Stress exposure causes an increase in Ca^2+^ followed by CaM interaction with the CaMBD, causing conformational changes that activate the transcriptional activity of CAMTA (Shen et al., 2015). The Ca^2+^/CaM-mediated activation demonstrates that CAMTA-CRE binding alone is not sufficient for transcription. The CRE must be occupied and the Ca^2+^ signal must be present.

In CRE engineering, this dual requirement is a benefit, not a limitation. Promoters with CGCG/CaRE motifs will only activate expression when both the CAMTAs are present and Ca^2+^ levels are elevated. This ensures that stress is inherently built in. Synthetic promoters constructed using CGCG/ABRE-CE modules will respond to stress-triggered Ca^2+^ signals autonomously, rather than constitutively active, thereby lowering the metabolic costs of transgene expression under non-stress conditions.

### 3.4 Evolutionary conservation of CAMTA-CRE recognition across eukaryotes: mechanistic insights from the animal kingdom

The CAMTA protein family is evolutionarily conserved among eukaryotes, with two members, CAMTA1 and CAMTA2, which were identified in the human genome and predominantly expressed in the heart and brain (Liu and Olson, 2006). This conservation transcends phylogenetics and the structural and molecular processes governing CAMTA-CRE recognition are preserved throughout eukaryotes, particularly the sequence-specific binding of the CG-1 domain to CGCG-core elements and its Ca^2+^/CaM-gated activation (Vuong-Brender et al., 2021). Insights gained from the biology of animal CAMTA protein are directly applicable to understanding and designing CAMTA-CRE interactions in agricultural systems.

The mammalian cardiac system is an excellent model for understanding the mechanism of CAMTA-CRE co-activators. CAMTA2 directly activates the homeobox transcription factor Nkx2–5 by binding to the promoter of the atrial natriuretic factor (ANF) gene, a CRE module that controls hypertrophic signaling and embryonic heart development (Song et al., 2006; Liu and Olson, 2006). This interaction demonstrates that the CAMTA CG-1 domain functions both as a standalone DNA-binding entity and as a scaffold for the assembly of combinatorial transcription factors at composite CRE modules. This is a principle that is directly reflected in plant systems, where CAMTA co-occupies CGCG/ABRE-CE promoter modules with bZIP and WRKY factors to control stress-gene activation (Saidi and Hajibarat, 2021; Gain et al., 2022). Mice with *CAMTA2* mutations exhibit compromised cardiac development, whereas reduced *CAMTA1* levels lead to cardiac anomalies in embryos (Song et al., 2006; Schwartz and Schneider, 2006), underscoring the necessity of precise CAMTA dosage at CRE modules for developmental gene programs. This principle is equally relevant in the design of CAMTA-responsive CREs in crops, where expression levels must be carefully calibrated controlled to avoid pleiotropic effects.

Invertebrate models further illuminate comprehension of the conservation of Ca^2+^/CaM-CAMTA regulatory mechanisms. In *Caenorhabditis elegans*, the broadly expressed CAMTA protein CAMT-1 interacts with multiple sites in promoter of the CaM. CaM subsequently regulates its own expression through a feedback mechanism reliant on CaM-binding sites on CAMT-1, thereby creating a self-regulating loop for Ca^2+^ homeostasis (Vuong-Brender et al., 2021). This feedback architecture, demonstrates an inherent autoregulatory characteristic of CAMTA-CRE system, with significant implications for the engineering of stress-response circuits in plants. Synthetic CAMTA-responsive promoters driving CaM or CaM-like genes could establish similar self-tuning Ca^2+^ signaling buffers in stress-affected crops.

In *Drosophila melanogaster*, dCAMTA regulates photoreceptor gene expression by binding to CREs in the rhodopsin gene promoter, while mutations in dCAMTA disrupt phototransduction termination, and dimerization via the CG-1 domain is crucial for nuclear localization and target gene activation (Han et al., 2006; Gong et al., 2007). The requirement for CG-1-mediated dimerization at CREs in *Drosophila* underscores a conserved structural necessity for tandem or closely positioned CGCG elements to accommodate the CAMTA dimer footprint, which is a design principle that should be incorporated into synthetic plant promoters employing CAMTA-responsive CRE modules. These cross-kingdom investigations collectively confirm that the mechanistic principles governing CAMTA-CRE recognition are highly conserved and validate the use of animal structural data to improve plant CRE engineering.

## 4 CAMTA-associated CREs in crop stress-responsive regulation

**Figure 1:**
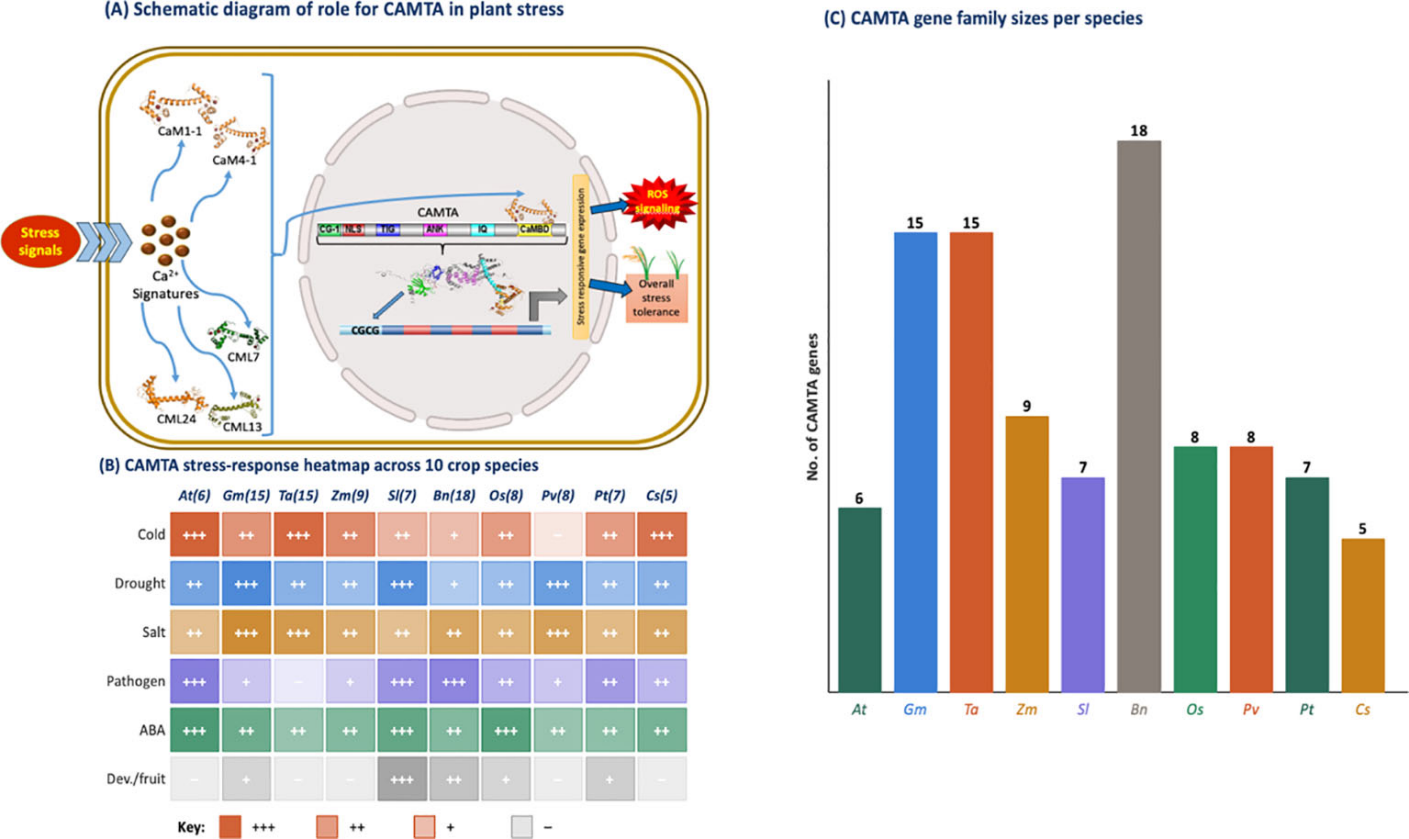
Diversity of CAMTA gene families and CGCG-core CRE distribution across major crop species. **(A)** Schematic diagram of role of CAMTA in plant stress; **(B)** Heatmap depicting the prevalence of CGCG-core elements in the promoters of CAMTA target genes across several stress conditions (cold, drought, salt and pathogen); **(C)** Graphical respresentation of CAMTA paralogs from *Arabidopsis thaliana* (6 members), *Glycine max* (15), *Triticum aestivum* (15), *Zea mays* (9), Solanum lycopersicum (7), and *Brassica napus* (18), with stress-response annotations. Colour intensity indicates the documented response strength, with +++ indicating extremely strong, ++ representing moderate, + signifying weak or fewer members, and – indicating not reported.

The stress-responsive CRE landscape in CAMTA-regulated promoters across major crop species is surveyed in this section, organized by stress type. The key CRE modules involved in each category are identified, along with the crops in which they operate and their potential as engineering targets (**Figure 1**).

### 4.1 Cold stress: CGCG-core elements and the CBF regulation

Cold stress illustrates the most mechanistically characterized link between CAMTA and CRE. In *Arabidopsis thaliana*, AtCAMTA3 and its partners AtCAMTA1/2 rapidly bind to CGCG-core elements in the promoters of *CBF1*, *CBF2*, and *ZAT12* upon cold exposure, before the canonical ICE1-CBF pathway is fully activated (Doherty et al., 2009). This places CAMTA-CRE interactions at the apex of the cold-acclimation transcriptional cascade. The CBF1 promoter contains multiple functional CGCG-core elements that collectively enhance cold inducibility (Zhou et al., 2025). Natural variation in CGCG copy number and sequence context among *Arabidopsis* variants is associated with differing degrees of cold tolerance (Kidokoro et al., 2017). All fifteen *TaCAMTA* genes in wheat (*Triticum aestivum*) exhibit a response to cold stress. *TaCAMTA1-A* and *TaCAMTA1-D* consistently show higher level expression under various forms of abiotic stress (Yang et al., 2020a). Cold stress in maize (*Zea mays*) triggers the overexpression of *ZmCAMTA4a*, *ZmCAMTA7a*, and *ZmCAMTA7b*, indicating that CGCG-containing promoters of downstream cold-responsive genes represent viable targets for engineering chilling-tolerant maize cultivars (Yue et al., 2015). Cold and salt stress upregulate one strawberry (*Fragaria × ananassa*) *CAMTA*, *FaCAMTA3*, whereas *FaCAMTA4* responds to cold, heat, and ethylene, reflecting broad CGCG-core and ABRE-CE CRE module activation in this soft fruit (Leng et al., 2015). Eleven *PeCAMTA* from moso bamboo (*Phyllostachys edulis*) genes exhibited peak expression at cold stress, with *PeCAMTA03* and *PeCAMTA06* having the highest transcript levels, indicating a transitory Ca²^+^/CAMTA-driven burst via CGCG-core CRE occupancy (Liu and Tang, 2023). Cold stress in *Oryza sativa* (rice) activates *OsCAMTA3b* and *OsCAMTA7b* while inhibiting *OsCAMTA1* and *OsCAMTA3a*, indicating that the CGCG-core CRE configurations in the target promoters of these paralogs are designed to respond to Ca²^+^ signature amplitudes produced by cold stimuli (Gain et al., 2024). *SmCAMTA2* and *SmCAMTA6* sustain prolonged cold exposure among all the *CAMTA*s isolated from eggplant (*Solanum melongena*), indicating that their downstream target promoters possess CGCG arrays with enhanced Ca²^+^/CAMTA binding affinity or increased chromatin accessibility during extended chilling (Cai et al., 2024). *BdCAMTA3* exhibits the most significant cold induction among the seven *CAMTA* family members of *Brachypodium distachyon*, correlating with the highest concentration of CGCG-core CRE modules in its downstream targets. In contrast, *BdCAMTA1* and *BdCAMTA6* are uniquely co-induced by cold, salt, and drought, rendering them valuable models for the design of multi-stress-responsive CRE modules in temperate cereals (Akbudak et al., 2024). Both *LchiCAMTA* genes from *Liriodendron chinense* (Chinese tulip tree) demonstrate a biphasic cold-stress expression profile characterized by an initial increase, a temporary decrease, and a subsequent resurgence. This pattern aligns with the phased activation of CGCG-core CRE modules, which initially respond to the Ca²^+^ surge at the onset of cold and are then reactivated by secondary downstream Ca²^+^ signaling during prolonged low-temperature exposure (Hong et al., 2024). Cold exposure activates different peach (*Prunus persica*) *CAMTA*s, *PpCAMTA1*, *PpCAMTA3*, and *PpCAMTA4*, with the latter two reaching their maximum expression one day prior to a decrease during prolonged cold storage, reflecting the transient Ca²^+^/CAMTA transcriptional surge observed in *Arabidopsis* (Yang et al., 2022a). In *Camellia sinensis*, cold acclimatization induces *CsCAMTA2*, *CsCAMTA3*, and *CsCAMTA5*, while de-acclimation reinstates the expression of *CsCAMTA4* and *CsCAMTA5* (Zhou et al., 2022; Zaman et al., 2022). The fluctuation of *CAMTA* expression during acclimatization and de-acclimation indicates that CGCG-dependent promoter modules will serve as transcriptional memory nodes, with direct implications for the engineering of stress memory in crops through CRE modification.

Base editing of CGCG arrays in the promoters of *CBF* homologs in cold-sensitive crops such as tropical maize and tomato is a documented engineering strategy expected to lower the Ca^2+^/CAMTA activation threshold, thereby enhancing cold-responsive expression and increasing chilling tolerance without the growth penalties associated with constitutive CBF overexpression (Kasuga et al., 1999).

### 4.2 Drought and ABA signalling: ABRE-CE and CGCG modules as dual targets

Drought stress induces the accumulation of ABA, which subsequently activates bZIP transcription factors that bind to ABREs (CACGTG). Simultaneously, Ca^2+^ signals activate CAMTA, which associates with ABRE-CE [(C/A)ACGCG(T/G/C)], a coupling element that requires occupancy by a canonical ABRE for optimal functionality (Finkler et al., 2007). The CAMTA-ABRE-CE relationship represents a convergence of ABA and Ca^2+^ signaling at a singular promoter module. Dual-responsive CRE clusters are therefore prime engineering candidates for enhancing drought tolerance. *AtCAMTA1* transiently regulates the expression of ABA-responsive genes in *Arabidopsis* during drought conditions by utilizing ABRE-containing promoters to integrate signals from membrane integrity and photosynthetic stress (Pandey et al., 2013). In soybean (*Glycine max*), all 15 *GmCAMTA* genes are up-regulated in response to 200 mM NaCl and PEG-induced dehydration, with *GmCAMTA8* demonstrating maximal expression under saline conditions (Wang et al., 2015). In common bean (*Phaseolus vulgaris*), *PhavuCAMTA1* expression significantly escalates under moderate drought stress, especially in tolerant genotypes (Saeidi et al., 2019). In *Musa acuminata* (banana), *MuCAMTA1*, *MuCAMTA3*, *MuCAMTA4*, and *MuCAMTA5* are upregulated under drought conditions, although *MuCAMTA2* shows no response (Meer et al., 2019). *HmCAMTA2* is the most significantly drought-responsive paralog from *Heimia myrtifoli*, exhibiting peak expression at 5 - 15% soil moisture, whereas *HmCAMTA3* and *HmCAMTA4* are down-regulated. This indicates that these paralogs function within opposing cis-regulatory element modules, representing distinct targets for modulating the drought-response threshold in this crop (Yang et al., 2022b). In *Chenopodium quinoa* (quinoa), six of the seven *CqCAMTA* genes exhibit significant up-regulation in response to PEG-induced water stress, demonstrating a wave-like activation profile of CRE that aligns with the sequential activation of ABRE-CE followed by CGCG modules as osmotic stress escalates, with *CqCAMTA03* displaying the most consistent root-specific up-regulation throughout the entire stress duration (Zhu et al., 2022a). *SiCAMTA2* and *SiCAMTA5* from *Sesamum indicum* (sesame) are upregulated in drought-resistant genotypes during water scarcity, indicating the active involvement of the ABRE-CE CRE module in drought tolerance (Kumar et al., 2024). All seventeen *PbCAMTA* genes from *Phoebe bournei* exhibit up-regulation in drought conditions, with *PbCAMTA1* showing the most significant increase (Zheng et al., 2024). Seven *CAMTA* genes (*CaCAMTA1–7*) were identified and characterized in chickpea (*Cicer arietinum*), demonstrating conserved domain architecture, varied expression throughout developmental stages, and upregulation in response to drought and ABA stress, with their promoters enriched in CGCG-core and ABRE cis-elements. A regulon of 1660 putative target genes with CGCG motifs were identified, indicating the role of *CaCAMTA*s in mediating abiotic stress tolerance and seed development in this significant legume crop (Sonkar et al., 2025).

Experimental evidence in soybean confirms ABRE-CE as a critical target for genetic engineering under drought stimuli. Overexpression of *GmCAMTA12* in *Arabidopsis* and soybean enhanced drought tolerance by improving water-use efficiency and activating stress-responsive gene networks (Noman et al., 2019). These findings validate that augmenting interaction strength of CAMTA-CRE at ABRE-CE modules is directly translatable to enhance drought tolerance. To increase ABRE module density in wheat, soybean, or maize, prime editing of the promoters of drought-responsive CAMTA target genes, including RD29A and NCED orthologs, constitutes a well-grounded, DSB-free strategy for enhancing drought resilience.

### 4.3 Salt stress: CRE signatures across crops

Salt stress activates CAMTA-CRE interactions through osmotic and ionic factors, leading to Ca^2+^ oscillations that increase CAMTA binding to CGCG and ABRE-CE motifs in salt-responsive promoters. *AtCAMTA3* mitigates salt stress in *Arabidopsis*, while *AtCAMTA6* modulates salt sensitivity during germination and seedling development (Rahman et al., 2016a). The CAMTA6-ABRE interaction exemplifies that a single CRE module can operate as a hub for both Ca^2+^ and ABA signaling under salt stress, a regulatory concept emphasized in the wider context of salt-inducible promoter engineering (Ding et al., 2026). The differing roles of several CAMTA family members, enabled by their unique CRE occupancy, highlight the importance of paralogue-specific CRE targeting in the development of salt tolerance. In wheat, NaCl stress induces activation of *TaCAMTA1-A*, *TaCAMTA1-D*, *TaCAMTA5-A*, *TaCAMTA5-D*, and *TaCAMTA6-B*, while suppressing several other *TaCAMTA* genes (Yang et al., 2020a). In pumpkin (*Cucurbita moschata* and *Cucurbita maxima*), salt stress differentially affects the expression of *CmoCAMTA* and *CmaCAMTA* in vascular and mesophyll tissues, implying that distinct CRE structures regulate tissue-specific salt responses (Yuan et al., 2021). In citrus (*Citrus sinensis* and *C. clementina*), *CitCAMTA1*, *CitCAMTA5*, and *CitCAMTA9* are significantly elevated under salt stress, while *CitCAMTA3* is suppressed. The promoter of the latter gene presumably lacks the cooperative CGCG/ABRE-CE modules seen in the salt-induced paralogs (Zhang et al., 2019). Six *Pvul-CAMTA* genes are augmented by salt stress in *Phaseolus vulgaris* (common bean) leaf tissue, where *PvUL-CAMTA-7* is down-regulated. Root-specific expressions are restricted to only *Pvul-CAMTA-1*, *Pvul-CAMTA-2* and *Pvul-CAMTA-4*, which shows organ-specific accessibility of the ABRE-CE/CGCG CRE module and provide a tissue-specific map of CAMTA-promoter targets for engineering salt tolerance in legumes (Büyük et al., 2019). Among the twenty *AsCAMTA* genes from *Avena sativa* (oat), *AsCAMTA5*, *AsCAMTA7*, and *AsCAMTA*19 exhibit significant up-regulation in response to salt stress, whereas *AsCAMTA7* and *AsCAMTA11* are responsive to drought. The dual stress responsiveness of *AsCAMTA7* aligns with the presence of shared CGCG-core and ABRE-CE cis-regulatory elements that react to the common Ca²^+^ and ABA signals associated with osmotic stress (Yang et al., 2024). Cross-species conservation analysis of CGCG-core and ABRE element distribution in CAMTA-regulated promoters is expected to yield universal CRE signatures for salt tolerance applicable to other crops.

### 4.4 Other abiotic stresses: hormone-responsive and heavy-metal CRE signatures

Beyond the primary abiotic stress categories, CAMTA-CRE interactions also facilitate responses to phytohormones, heavy metals, and UV radiation, indicating a comprehensive cis-regulatory network that synthesizes many abiotic signals. In *Arabidopsis*, *AtCML24* interacts with *AtCAMTA2* to augment aluminum-activated malate transporter1 (*AtALMT1*)-mediated malate exudation and aluminium (Al) resistance where the AtCML24-AtCAMTA2 complex achieves the augmentation by repressing *AtWRKY46*, a negative regulator of *ALMT1* expression, thereby overcoming Al-induced root growth inhibition (Tokizawa et al., 2015; Zhu et al., 2022b). The hormonal sensitivity of CAMTA-CRE interactions has been thoroughly obsereved in these crop species. In maize, expression profiling of nine *ZmCAMTA* genes under multiple phytohormone treatments revealed distinct CRE-dependent regulatory patterns (Yue et al., 2015). SA stimulated *ZmCAMTA3* in both leaf and root tissues, while IAA up-regulated *ZmCAMTA5* and *ZmCAMTA7b* in roots and *ZmCAMTA7a* in leaves. ABA induced *ZmCAMTA3*, *4b*, *6*, *7a*, and *7b* in leaves and *ZmCAMTA7b* in roots and MeJA elevated *ZmCAMTA4a*, *7a*, *1*, and *6* in roots and shoots (Yue et al., 2015). The promoters of these differentially regulated *ZmCAMTA* target genes are enriched with ABRE, ABRE-CE, and CGCG motifs, confirming that hormone-triggered Ca^2+^ signals are decoded through CRE occupancy. Moreover, CRE in CAMTA promoter are also found to play crucial role in hormonal regulation and downstream signaling. In poplar (*Populus trichocarpa*), *PtCAMTA* genes respond differentially to different treatments of ABA, SA and MeJA. Short-term ABA treatment elevated *PtCAMTA1*, *2*, *3*, *6*, and *7* transcripts in roots, while SA suppressed all root-expressed *PtCAMTA* genes but induced *PtCAMTA1*, *5*, and *7* in leaves (Wei et al., 2017). UV-B treatment reduced *PpCAMTA1* expression in peach, while MeJA suppressed *PpCAMTA3* and modestly elevated *PpCAMTA4* and *5* (Yang et al., 2022a). In tobacco (*Nicotiana tabacum*), cadmium (Cd) stress induced *NtabCAMTA* genes in leaf and root tissues, with *NtabCAMTA6* specifically down-regulated in roots under Cd exposure (Kakar et al., 2018). The hormone- and heavy metal-responsive expression of *NtabCAMTA* genes reflects the activity of ABRE-CE and CGCG elements in their target promoters in response to non-canonical Ca^2+^ signals. In cucumber (*Cucumis sativus*), *CsCAMTA1* expression increased after 3 h of IAA or MeJA treatment and after 6 h of ABA exposure, whereas *CsCAMTA3* and *4* were up-regulated after 6 h of IAA and reached peak expression at 48 h post-MeJA treatment (Gao et al., 2021). *CsCAMTA4* showed down-regulation under short-term ABA treatment and *CsCAMTA1*, *3*, and *4* expressions gradually rose after 48 h under salt and drought stress, whereas *CsCAMTA2* was transiently suppressed (Gao et al., 2021). Collectively, these datasets reveal that CGCG-core and ABRE-CE elements in CAMTA-regulated promoters function as convergence nodes for Ca^2+^ signals arising from diverse abiotic inputs beyond cold, drought, and salinity. The paralogue-specific and tissue-specific CRE occupancy patterns observed across hormones and heavy metals identify these promoter modules as candidates for precision base editing or prime editing to fine-tune CAMTA-dependent transcriptional responses to multiple abiotic cues simultaneously.

### 4.5 Biotic stress: CRE modules in defense gene promoters

In *Arabidopsis*, CAMTA3/SR1 serves as a principal negative regulator of plant immunity. It attaches to CGCG-core areas in the *EDS1* promoter and inhibits salicylic acid (SA)-mediated defense (Pandey et al., 2013; Rahman et al., 2016a). The *EDS1* promoter contains a functional CGCG element regulated by CAMTA, which maintains immune system equilibrium in the absence of pathogen attack. During pathogen invasion, Ca^2+^ spikes alter CAMTA activity, relieving *EDS1* repression and facilitating defense mediated by salicylic acid bursts. Modifying this CGCG element to reduce CAMTA affinity represents a strategy to basal immunity, though the associated fitness costs require careful evaluation. In tomato (*Solanum lycopersicum*), *SlSR1* and *SlSR3L* function as negative regulators of defense against *Botrytis cinerea* and *Pseudomonas syringae*, and silencing these genes enhanced resistance (Li et al., 2014). Modifying the CGCG-containing promoter elements at the CRE level that regulate the *SlSR1* and *SlSR3L* target genes would attain the same derepression with greater precision and without disrupting the entire CAMTA regulon. Eighteen *BnCAMTA* genes in oilseed rape (*Brassica napus*) exhibit responsiveness to the pathogen *Sclerotinia sclerotiorum*, its oxalic acid toxin, and the signaling molecules of SA and JA (Rahman et al., 2016b). The promoters of *BnCAMTA*-regulated genes that react to SA and JA contain both W-box elements and CGCG motifs, indicating that combinatorial CAMTA-WRKY CRE modules regulate Brassica defensive responses. In response to *Alternaria alternata* infection, *PtCAMTA1* and *PtCAMTA2* from *Populus trichocarpa* (poplar) are upregulated, whereas most of the genes are downregulated. This pattern aligns with a biotic-stress CRE reconfiguration and presents dual-target CRE editing prospects for enhanced disease resistance in poplar (Wei et al., 2017).

Experimental evidence in rice further substantiates the connection between CAMTA and biotic stress at the CRE level. Partial T-DNA knockdown of one rice *CAMTA* gene, *OsCBT* conferred broad-spectrum resistance to rice neck blast fungus (*Magnaporthe grisea*) and bacterial leaf blight (*X. oryzae* pv. *oryzae*), accompanied by constitutive PR gene expression and hypersensitive response induction (Koo et al., 2009). Subsequent transcriptomic profiling identified 281 differentially expressed genes in the *oscbt-1* mutant, with strong enrichment for NBS-LRR resistance proteins and PR-related genes under pathogen-free conditions (Chung et al., 2020). These findings established *OsCBT* as a negative regulator of plant immunity in rice and directly implicate defense signaling through extensive transcriptional reprogramming of CGCG-regulated immune gene networks. The CGCG elements in the promoters of *OsCBT* target genes therefore represent validated CRE targets for editing strategies aimed at broadening pathogen resistance in rice and related cereals. *MeCAMTA3* from *Manihot esculenta* (cassava) functions as a negative regulator of resistance to cassava bacterial blight, as evidenced by trangenically silenced plants exhibiting enhanced resistance compared to overexpression lines. This confirms that *MeCAMTA3* CGCG-CRE repression of defense gene promoters attenuates SA-mediated immunity, paralleling the mechanistic rationale of AtCAMTA3-EDS1 in *Arabidopsis* (Chang et al., 2020).

## 5 Genome editing of CREs: CRISPR-based strategies for crop improvement

**Figure 2:**
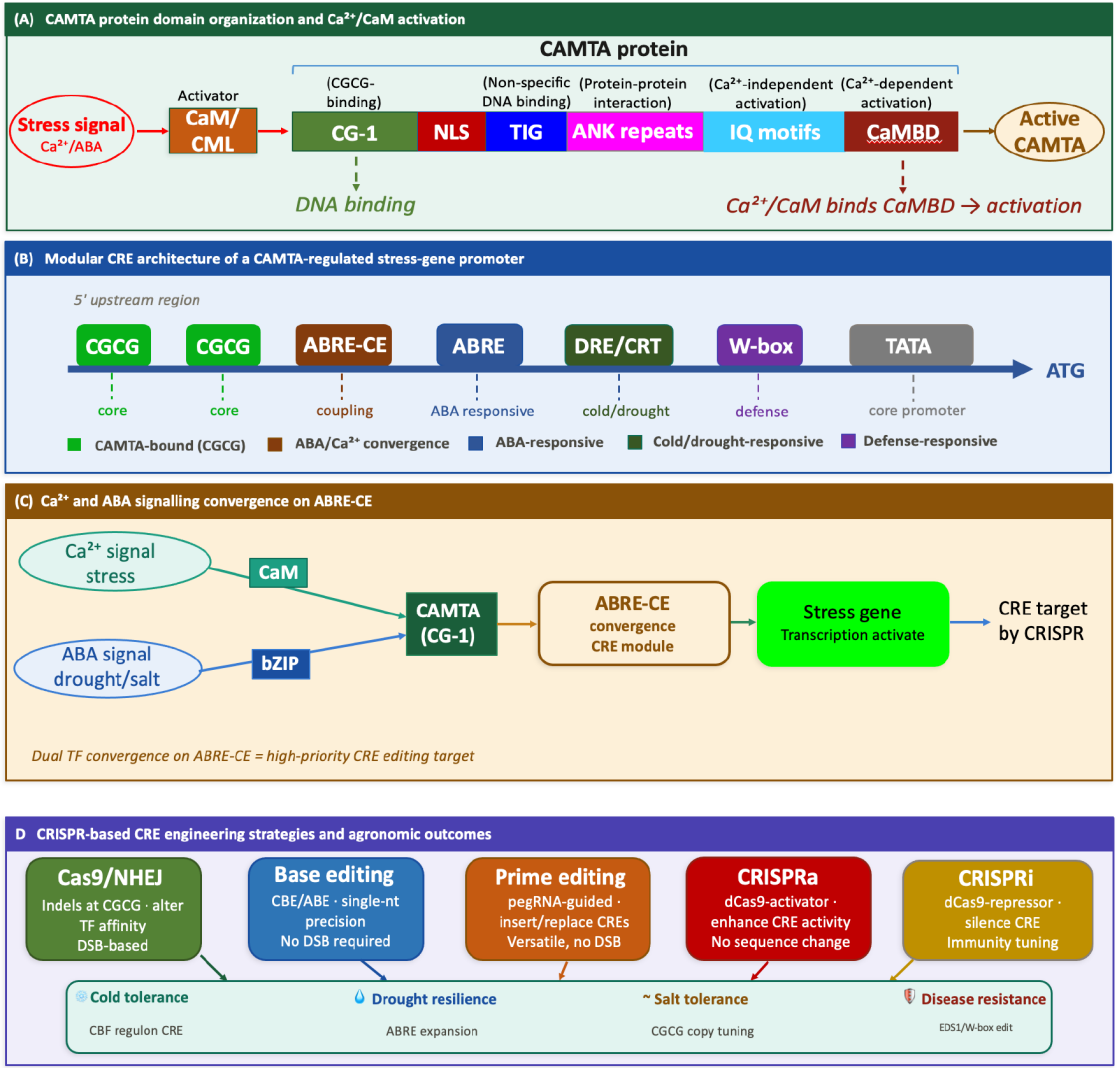
Schematic overview of CAMTA-CRE interactions and CRISPR-based engineering strategies. **(A)** Domain organization of CAMTA proteins: CG-1 (DNA-binding, CGCG-core recognition), TIG (cooperative DNA binding), ANK repeats (protein-protein interaction), IQ motifs (Ca^2+^-independent CaM binding), and CaMBD (Ca^2+^-dependent CaM activation). **(B)** Promoter architecture of a typical CAMTA-regulated stress gene, illustrating CGCG-core elements, ABRE-CE, canonical ABRE, DRE/CRT element, W-box, and TATA box. **(C)** Integration of Ca^2+^ signaling (via CaM-CAMTA activation) and ABA signaling (via bZIP-ABRE binding) within the ABRE-CE module. **(D)** CRISPR engineering methodologies for CRE modification include Cas9-induced indels, base editing (CBE/ABE), prime editing (pegRNA-guided insertion/substitution), CRISPRa (dCas9-activator for augmented CRE activity), and CRISPRi (dCas9-repressor for CRE silencing). The agronomic outcomes of CRE engineering encompass enhanced cold tolerance, drought resilience, salt tolerance, and disease resistance in modified crops.

The mechanistic understanding of CAMTA-CRE interactions outlined above provides a foundation for accurate CRE engineering. CRISPR/Cas technologies offer various modalities for CRE modification, each exhibiting distinct advantages suited to certain engineering goals (**Figure 2**).

### 5.1 CRISPR/Cas9 nuclease-based CRE editing

The traditional CRISPR/Cas9 method induces DSBs at specific loci, repaired by either non-homologous end joining (NHEJ) or homology-directed repair (HDR) (Yang et al., 2020b). In CRE editing, Cas9-mediated NHEJ at CGCG or ABRE-CE elements may result in minor insertions or deletions (indels) that alter the binding affinity of transcription factors, analogous to the modifications seen in natural promoters. Rodríguez-Leal et al. (2017) demonstrated this technique in tomatoes by altering the promoter of CLAVATA3 to provide a quantifiable range of fruit size variation, which is an outstanding proof-of-concept for CRE editing in agricultural improvement.

Editing CGCG-core elements in the promoters of *CBF* genes or *EDS1* orthologs in crops using Cas9 is a direct method to modify CAMTA-dependent cold or defense responses. Employing sgRNA arrays for multiplexed editing should simultaneously alter several CGCG elements within a stress regulon, resulting in additive enhancements in tolerance.

### 5.2 Base editing: precise single-nucleotide CRE modification

Base editors (adenine base editors [ABEs] and cytosine base editors [CBEs]) enable precise single-nucleotide changes within CREs without DSBs or HDR templates, making them ideal for fine-tuning CRE function (Komor et al., 2016; Gaudelli et al., 2017). In a CGCG-core element, the consensus sequence is shifted from the classic CAMTA-binding sequence to variants with different affinities by changing one C to T or A to G. Base editing enables of the CAMTA-CRE interaction intensity without completely abolishing transcriptional regulation.

Base editing of ABRE elements is especially promising for drought engineering. Changing the nucleotides surrounding ABRE-CE [(C/A)ACGCG(T/G/C)] usually alter the element from Ca^2+^/CAMTA-primary to bZIP-primary recognition, or create new dual-recognized sequences that expand the regulatory repertoire. The DSB-free nature of base editing also confers regulatory advantages in jurisdictions requiring non-transgenic modifications.

### 5.3 Prime editing: versatile CRE rearrangement

Prime editing utilizes a Cas9 nickase linked to reverse transcriptase and a prime editing guide RNA (pegRNA) to implement specified sequence alterations such as substitutions, insertions, or deletions, at a designated locus without causing double-strand breaks (Anzalone et al., 2019). In CRE engineering, prime editing facilitates: (i) augmentation of CGCG or ABRE motifs to enhance stress-responsive expression; (ii) meticulous modification of the interstitial regions between CRE modules to optimize cooperative transcription factor binding; and (iii) substitution of inferior CRE variants with high-affinity CAMTA-binding sequences identified via *in vitro* selection.

Prime editing of promoter CREs in CAMTA-regulated stress genes offers the possibility of developing crops with pre-optimized stress-response modules, effectively incorporating the regulatory architecture of stress-adapted wild relatives into elite cultivars free from linkage drag.

### 5.4 CRISPRa and CRISPRi: epigenetic modulation of CAMTA-responsive CREs

CRISPR activation (CRISPRa) utilizes a catalytically inactive Cas9 (dCas9) connected to transcriptional activators targeting CREs, enhancing promoter activity without altering the DNA sequence. CRISPR interference (CRISPRi) employs dCas9 and repressors to deactivate target CREs (Morelli et al., 2021). CRISPRa deployed at CAMTA-regulated stress gene promoters enhance the efficacy of the CGCG/ABRE-CE module in stress-affected crops, while CRISPRi can relieve CAMTA-mediated suppression of genes related to immunity in pathogen-affected plants.

Inducible dCas9 systems, whose guide RNA synthesis is controlled by stress-responsive promoters, have the capacity to create self-amplifying CRE-editing loops, in which initial stress signals trigger further overexpression of stress-responsive CRE modules. This circuit logic has been verified in mammalian cells (Zhu et al., 2020) and represents an innovative application for plant stress engineering.

### 5.5 Synthetic promoter design using CAMTA-responsive CRE modules

Synthetic biology approaches will generate new promoters from defined CRE constructs, in addition to modifying endogenous promoters. Synthetic promoters have been engineered to facilitate stress-specific, high-amplitude gene expression by integrating optimal CGCG-core arrays, adjacent ABRE-CE elements, and minimal TATA-box cores, thereby avoiding the pleiotropic effects associated with complete endogenous promoters (Rushton et al., 2002; Bhullar et al., 2003).

The molecular insights into CAMTA-CRE interactions elucidated in this review provide a framework for the development of Ca^2+^/stress-responsive synthetic promoters. The main design principles are: (i) including at least two tandem CGCG-core elements separated by optimal spacers for the TIG domain; (ii) placing at least one ABRE-CE adjacent to a canonical ABRE to capture ABA/Ca^2+^ convergence signaling; (iii) incorporating stress-specific silencer elements to prevent leaky expression in the absence of stress; and (iv) adding tissue-specific enhancer modules for targeted organ-level expression in roots (drought response) or leaves (cold response).

Design concepts of synthetic stress-inducible promoters, including tandem CRE repeats, modular combinatorial assembly, tissue-specific elements, and bidirectional designs, are an active frontier in precision agricultural engineering (Ding et al., 2026). Synthetic CAMTA-responsive promoters are designed to precisely drive stress-tolerance genes (e.g., *DREB1A*, or *AVP1*) in commercial crop varieties, resulting in crops that exhibit robust, well-timed defensive responses without incurring inherent fitness penalties.

## 6 CAMTA-CRE interactions in crop development: opportunities for quality trait engineering

Beyond stress tolerance, CAMTA-regulated promoters control developmental processes, particularly fruit ripening and senescence, which directly influence crop quality. The first *CAMTA* gene characterized, *NtER1* in tobacco, was identified as an ethylene-upregulated regulator of senescence, functioning by binding to CGCG-containing promoter regions in a Ca^2+^/CaM-dependent manner (Yang and Poovaiah, 2002). This seminal finding indicates that CRE editing in the promoters of ripening genes represents a tractable strategy for extending the shelf life of fruit. Seven *SlSR* (*CAMTA*) genes in tomatoes exhibit stage-specific expression throughout fruit development and ripening. Conversely, *SlSR4* is specifically expressed in orange and ripening fruit tissue (Yang et al., 2012). The *SlSR4* promoter is a tissue-specific CRE module active during the ripening process and represents a precision target for fruit quality improvement. The overexpression of *PbrCAMTA2* in pear (*Pyrus bretschneideri*) enhanced fruit firmness and delayed ripening by regulating the expression of cell-wall genes and the ethylene biosynthesis enzyme ACO (Yu et al., 2023), identifying the promoters of *TBG*, *XET*, and *ACO* as prospective candidates for CAMTA-CRE engineering to improve shelf life. In *Gossypium hirsutum*, *GhCAMTA2A.2* and *GhCAMTA7A* are specifically expressed during fiber initiation and secondary cell wall synthesis, correlating with fiber strength (Pant et al., 2018). The CRE architecture regulating these *GhCAMTA* genes, and their associated fibre-development targets is a largely unexplored area for precision CRE editing aimed at improving cotton fibre quality without the introduction of transgenes. The overexpression of *PpyCAMTA2* from *Pyrus pyrifolia* (Asian pear) postpones fruit ripening and increases firmness in both pear and transgenic tomato by repressing ethylene biosynthesis gene promoters through a DNA methylation-dependent mechanism at CGCG-core elements, thereby introducing an epigenetic aspect to CAMTA-CRE regulation beyond the Ca²^+^/CaM-dependent binding (Song et al., 2024). The elevation of calmodulin induced by CaCl_2_ activates the EjCaM7–EjCAMTA3 interaction, allowing *EjCAMTA3* to bind to CGCG-core elements in the promoters of lignin biosynthesis genes in *Eriobotrya japonica* (loquat) plant, which reduces lignin accumulation during cold storage and mitigates chilling injury. This identifies the CGCG-containing promoters as targets for CRE editing to preserve fruit quality during cold storage (Hou et al., 2022). The Ca²^+^-dependent DcCaM7–DcCAMTA6 complex enhances the transcription of GABA shunt genes via CRE occupancy at their promoters, increasing GABA levels that facilitate stress tolerance in fresh-cut carrots (*Daucus carota*) during storage and positioning *DcCAMTA6* target gene promoters as candidates for CRE engineering aimed at extending post-harvest quality in root vegetables (Zhang et al., 2023).

The treatment of *Cucumis melo* (cantaloupe) with CaCl_2_ increases phenolic content and antioxidant activity in fresh-cut cantaloupe by facilitating CmCAMTA4-mediated binding to CGCG-core and ABRE-CE elements in the promoters of phenylpropanoid pathway genes, indicating that *CmCAMTA4* acts as a Ca²^+^-dependent transcriptional activator of gene networks that sustain quality under commercial cold storage conditions (You et al., 2024).

## 7 Regulatory and societal considerations for CRE-edited crops

### 7.1 Regulatory landscape: CRE editing as a non-transgenic approach

One of the primary advantages of CRE-based crop engineering is its alignment with contemporary regulations for genome-edited crops. Modifying endogenous CREs such as altering specific base pairs inside a promoter or adjusting the quantity of inherent cis-elements, results in alterations that fall within the spectrum of natural genomic variation and may occur via standard mutagenesis. This differs fundamentally from transgenic techniques that introduce exogenous coding sequences.

The SECURE rule of USDA APHIS (2021) in the United States stipulates that a plant is exempt from regulation if alterations to its genome could have been achieved by conventional breeding (Hoffman, 2021). This encompasses the majority of CRISPR-mediated CRE modifications, including minor insertions, deletions, or substitutions inside promoter regions. The proposed New Genomic Techniques (NGT) legislation in the European Union (2023, now under parliamentary consideration) aims to exclude Category 1 NGT plants from stringent GMO regulatory pathways (Schulman et al., 2025). In CAMTA-CRE engineering, alterations that modify CGCG-core elements by base editing or prime editing, without the incorporation of foreign DNA sequences, are expected to be exempt from regulation in several jurisdictions, significantly accelerating and reducing the cost of introducing CRE-edited stress-tolerant crops to the market compared to transgenic varieties.

### 7.2 Field performance and phenotypic stability

Increasing data from field testing demonstrates that CRE-edited crops have superior and stable performance. Modifications to tomato lines via promoter CRE alterations affecting fruit size and flowering time (Rodríguez-Leal et al., 2017; Zsögön et al., 2018) have shown that promoter modifications yield traits that are stable, heritable, and consistent across generations and environments. These precedents support the expectation that CAMTA-CRE-edited stress-tolerance traits will likewise exhibit enduring field performance.

Field evaluation of CAMTA-CRE-edited lines must assess the dynamics of stress-responsive gene expression under natural stress conditions, yield and agronomic performance in both stressed and non-stressed environments, reproductive fitness metrics to detect adverse effects from altered stress-signaling thresholds, and multi-year, multi-location trial data to analyze genotype-by-environment interactions of the CRE modifications.

## 8 Future perspectives

In the field of CAMTA-CRE biology, several research areas require strategic focus in the next years. A primary objective is to develop a CRE atlas for CAMTA target promoters through systematic chromatin immunoprecipitation sequencing (ChIP-seq) and ATAC-seq profiling. This comprehensive approach would provide datasets analogous to the mammalian ENCODE initiative, facilitating the identification of all CGCG-core and ABRE-CE elements appropriate for genome-wide editing.

Establishment of high-throughput CRE variant libraries through massively parallel reporter assays (MPRAs) would enable systematic evaluation of thousands of CRE variant sequences derived from targeted mutagenesis of CGCG areas, generating quantitative models linking CAMTA-binding affinities to gene expression outcomes. This would enhance CRE editing precision, enabling the achievement of designated expression levels rather than just binary on/off outcomes.

Development of multi-stress CRE modules is equally important, given that many crops encounter several environmental challenges concurrently. Engineering promoters capable of simultaneously responding to multiple stress signals, such as CGCG/Ca^2+^, ABRE/ABA, and DRE/DREB, would enable the development of crops resilient to diverse stressors through a single editing intervention.

Epigenome editing is an emerging discipline that emphasizes the significance of chromatin accessibility for the correct functioning of CREs. Techniques deploying dCas9 fused to histone acetyltransferases or DNA demethylases have been shown to activate target loci in plant systems (Morelli et al., 2021), and application of this approach to CAMTA-responsive CREs under stress conditions is a tractable avenue for enhancing the durationalong with the intensity of stress-responsive gene expression in crops that suppress these genes via epigenetic mechanisms.

AI-enhanced synthetic CRE design has significant potential. Machine learning models utilizing datasets of plant promoters to forecast gene expression from CRE sequences, combined with CAMTA-CRE binding data, enable the creation of entirely computer-designed synthetic stress-responsive promoters tailored to specific crops, stressors, and desired expression profiles.

Despite these opportunities, several challenges must be addressed before deploying CAMTA-centered cis-engineering at scale. Several CAMTA promoters and their downstream targets remain insufficiently characterized at high resolution. The function of motifs is contingent upon the environment and the activity of the same cis element vary based on the chromatin state or the adjacent sequences. Additional genome-wide binding and promoter-editing investigations in economically significant crop species are required. Future studies should integrate motif mining, chromatin profiling, transcriptomics, and genome editing into a unified experimental framework, with machine learning approaches to determine which CRE combinations will enhance stress resistance while maintaining yield.

## 9 Conclusion

Cis-regulatory elements in CAMTA-regulated promoters, specifically CGCG-core elements and ABRE-CE sequences, represent valuable targets for precision crop improvement against several stressors. The review has highlighted the mechanistic basis of CRISPR/Cas technologies, including nuclease editing, base editing, and epigenome editing, which precisely target this CRE landscape. The non-transgenic nature of many CRE editing methods is critical for navigating regulatory pathways, and evidence from field trials demonstrates that these modifications yield stable and viable crop phenotypes. Successfully translating insights into CRE-edited varieties requires advances in CRE atlas development, high-throughput variant evaluation, and clear communication with stakeholders. The integration of Ca^2+^ signaling, structural insights into CAMTA-CRE recognition, and precision genome editing presents a unique opportunity to develop stress-resilient crops that address global food security issues and Sustainable Development Goals related to combating climate change. By focusing on the cis-regulatory landscape through combined synthetic biology and genome editing approaches, CAMTA-associated cis-elements can be transformed into effective tools for crop enhancement while minimizing adverse effects on plant health.

## Glossary

ABA: abscisic acid
ABRE: ABA-responsive element
ABRE-CE: ABRE coupling element
ABE: adenine base editor
ACO: aminocyclopropane-1-Carboxylate Oxidase
ANK: ankyrin repeat domain
AtALMT1: aluminum-activated malate transporter1
AsCAMTA: *Avena sativa* CAMTA
AtCAMTA: *Arabidopsis thaliana* calmodulin-binding transcription activator
ATAC-seq: assay for transposase-accessible chromatin sequencing
AVP1: *Arabidopsis* vacuolar H^+^-pyrophosphatase 1
BdCAMTA3: *Brachypodium distachyon* CAMTA
BnCAMTA: *Brassica napus* CAMTA
Ca^2+^: calcium ions
CaCl₂: calcium chloride
CaM: calmodulin
CaMBD: calmodulin-binding domain
CaCAMTA: *Cicer arietinum* CAMTA
CAMTA: calmodulin-binding transcription activator
CaRE: Ca^2+^-responsive element
Cas: CRISPR-associated protein
CBE: cytosine base editor
CBF: C-repeat binding factor
ChIP-seq: chromatin immunoprecipitation sequencing
CitCAMTA: *Citrus* CAMTA
CmaCAMTA: *Cucurbita maxima* CAMTA
CML: calmodulin-like protein
CmoCAMTA: *Cucurbita moschata* CAMTA
CqCAMTA: *Chenopodium quinoa* CAMTA
CRISPR: clustered regularly interspaced short palindromic repeats
CRISPRa: CRISPR activation
CRISPRi: CRISPR interference
CsCAMTA: *Camellia sinensis* CAMTA
CmCAMTA: *Cucumis melo* CAMTA
dCAMTA: *Drosophila melanogaster* CAMTA
DcCaM7: *Daucus carota* calmodulin
dCas9: catalytically inactive Cas9
DNA: Deoxyribonucleic Acid
DREB: dehydration-responsive element-binding protein
DRE/CRT: dehydration-responsive element/C-repeat
DSB: double-strand break
EDS1: enhanced disease susceptibility 1
EjCaM: *Eriobotrya japonica* calmodulin
EjCAMTA: *Eriobotrya japonica* CAMTA
FaCAMTA: *Fragaria ×*
FEX1: Fluoride Export Gene 1
GhCAMTA: *Gossypium hirsutum* CAMTA
GmCAMTA: *Glycine max* CAMTA
GMO: Genetically Modified Organism
HDR: homology-directed repair
HmCAMTA2: *Heimia myrtifoli* CAMTA
ICE: inducer of CBF expression
Inr: Initiator
IQ: isoleucine-glutamine motif
JA: jasmonic acid
LchiCAMTA: *Liriodendron chinense* CAMTA
MeCAMTA: *Manihot esculenta* CAMTA
MeJA: methyl jasmonate
MPRA: massively parallel reporter assay
MuCAMTA: *Musa acuminata* CAMTA
NCED: 9-cis-epoxycarotenoid dioxygenase
NGT: new genomic techniques
NHEJ: non-homologous end joining
Nkx: NK Homeobox
NLS: nuclear localization signal
NtabCAMTA: *Nicotiana tabacum* CAMTA
NtER1: *Nicotiana tabacum* ethylene-responsive 1
OsCAMTA: *Oryza sativa* CAMTA
OsCBT: *Oryza sativa* calmodulin-binding transcription factor
PbCAMTA: *Phoebe bournei* CAMTA
PbrCAMTA: *Pyrus bretschneideri* CAMTA
PeCAMTA: *Phyllostachys edulis* CAMTA
PhavuCAMTA: *Phaseolus vulgaris* CAMTA
pegRNA: prime editing guide RNA
PpCAMTA: *Prunus persica* CAMTA
PpyCAMTA: *Pyrus pyrifolia* CAMTA
PtCAMTA: *Populus trichocarpa* CAMTA
Pvul-CAMTA: *Phaseolus vulgaris* CAMTA
qRT-PCR: quantitative reverse transcriptase PCR
RD29A: responsive to desiccation 29A
RNA: Ribonucleic Acid
RNAi: RNA interference
ROS: reactive oxygen species
SA: salicylic acid
sgRNA: single-guide RNA
SiCAMTA: *Sesamum indicum* CAMTA
SlSR: *Solanum lycopersicum* signal responsive
SmCAMTA: *Solanum melongena* CAMTA
SR: signal responsive
TaCAMTA: *Triticum aestivum* CAMTA
TBG: β-galactosidase
TIG: transcription factor immunoglobulin domain
TSS: transcription start site
USDA: United States Department of Agriculture
W-box: WRKY-binding box
WRKY: WRKY domain transcription factor
XET: xyloglucan endotransglucosylase
ZmCAMTA: *Zea mays* CAMTA.

## References

[B1] Abdel-Hameed A. A. Liao W. Prasad K. V. Reddy A. S. (2024). CAMTAs, a family of calmodulin-binding transcription factors, are versatile regulators of biotic and abiotic stress responses in plants. Crit. Rev. Plant Sci. 43, 171–210. doi: 10.1080/10407782.2024.2302671 37339054

[B2] Ahmar S. Gill R. A. Jung K. H. Faheem A. Qasim M. U. Mubeen M. . (2020). Conventional and molecular techniques from simple breeding to speed breeding in crop plants: recent advances and future outlook. Int. J. Mol. Sci. 21, 2590. doi: 10.3390/ijms21072590 32276445 PMC7177917

[B3] Akbudak M. A. Çetin D. Filiz E. Srivastava V. (2024). Genome-wide exploration and analysis of plant stress-responsive CAMTA transcription factor genes in Brachypodium distachyon and their expression patterns under environmental challenges. S. Afr. J. Bot. 166, 208–217. doi: 10.1016/j.sajb.2024.01.048 38826717

[B4] Anzalone A. V. Randolph P. B. Davis J. R. Sousa A. A. Koblan L. W. Levy J. M. . (2019). Search-and-replace genome editing without double-strand breaks or donor DNA. Nature 576, 149–157. doi: 10.1038/s41586-019-1711-4 31634902 PMC6907074

[B5] Bähler M. Rhoads A. (2002). Calmodulin signaling via the IQ motif. FEBS Lett. 513, 107–113. doi: 10.1016/S0014-5793(01)03239-2 11911888

[B6] Bhullar S. Chakravarthy S. Advani S. Datta S. Pental D. Burma P. K. (2003). Strategies for development of functionally equivalent promoters with minimum sequence homology for transgene expression in plants: cis-elements in a novel DNA context versus domain swapping. Plant Physiol. 132, 988–998. doi: 10.1104/pp.103.020602 12805627 PMC167037

[B7] Büyük İ. İlhan E. Şener D. Özsoy A. U. Aras S. (2019). Genome-wide identification of CAMTA gene family members in Phaseolus vulgaris L. and their expression profiling during salt stress. Mol. Biol. Rep. 46, 2721–2732. doi: 10.1007/s11033-019-04716-8 30843175

[B8] Cai P. Lan Y. Gong F. Li C. Xia F. Li Y. . (2024). Identification and molecular characterization of the CAMTA gene family in Solanaceae with a focus on the expression analysis of eggplant genes under cold stress. Int. J. Mol. Sci. 25, 2064. doi: 10.3390/ijms25042064 38396743 PMC10888690

[B9] Chang Y. Bai Y. Wei Y. Shi H. (2020). CAMTA3 negatively regulates disease resistance through modulating immune response and extensive transcriptional reprogramming in cassava. Tree Physiol. 40, 1520–1533. doi: 10.1093/treephys/tpaa093 32705122

[B10] Choi M. S. Kim M. C. Yoo J. H. Moon B. C. Koo S. C. Park B. O. . (2005). Isolation of a calmodulin-binding transcription factor from rice (Oryza sativa L.). J. Biol. Chem. 280, 40820–40831. doi: 10.1074/jbc.M504616200 16192280

[B11] Chung J. S. Koo S. C. Jin B. J. Baek D. Yeom S. I. Chun H. J. . (2020). Rice CaM-binding transcription factor (OsCBT) mediates defense signaling via transcriptional reprogramming. Plant Biotechnol. Rep. 14, 309–321. doi: 10.1007/s11816-020-00603-y 30311153

[B12] Cui Y. Cao Q. Li Y. He M. Liu X. (2023). Advances in cis-element-and natural variation-mediated transcriptional regulation and applications in gene editing of major crops. J. Exp. Bot. 74, 5441–5457. doi: 10.1093/jxb/erad248 37402253

[B13] Debnath T. Dhar D. G. Dhar P. (2024). Molecular switches in plant stress adaptation. Mol. Biol. Rep. 51, 20. doi: 10.1007/s11033-023-09051-7 38108912

[B14] Ding L. N. Sun Q. S. Li J. Y. Hu Y. H. Wang J. Tan X. L. . (2026). Molecular switches in plant stress adaptation: The function and engineering of inducible promoters. J. Agric. Food. Chem. 74, 13331–13348. doi: 10.1021/acs.jafc.5c12211 42012136

[B15] Doherty C. J. Van Buskirk H. A. Myers S. J. Thomashow M. F. (2009). Roles for Arabidopsis CAMTA transcription factors in cold-regulated gene expression and freezing tolerance. Plant Cell 21, 972–984. doi: 10.1105/tpc.108.063958 19270186 PMC2671710

[B16] Du L. Ali G. S. Simons K. A. Hou J. Yang T. Reddy A. S. N. . (2009). Ca^2+^/calmodulin regulates salicylic-acid-mediated plant immunity. Nature 457, 1154–1158. doi: 10.1038/nature07612 19122675

[B17] Fang H. Wang P. Ye F. Li J. Zhang M. Wang C. . (2022). Genome-wide identification and characterization of the calmodulin-binding transcription activator (CAMTA) gene family in plants and the expression pattern analysis of CAMTA3/SR1 in tomato under abiotic stress. Int. J. Mol. Sci. 23, 6264. doi: 10.3390/ijms23116264 35682943 PMC9181194

[B18] Finkler A. Ashery-Padan R. Fromm H. (2007). CAMTAs: calmodulin-binding transcription activators from plants to human. FEBS Lett. 581, 3893–3898. doi: 10.1016/j.febslet.2007.07.051 17689537

[B19] Gain H. De S. Banerjee J. (2024). Expressional vagaries of OsCAMTA genes under differential abiotic stresses supported with protein-protein interaction study and prediction of miRNA target sites. Plant Biotechnol. Rep. 18, 637–658. doi: 10.1007/s11816-024-00899-0 30311153

[B20] Gain H. Nandi D. Kumari D. Das A. Dasgupta S. B. Banerjee J. (2022). Genome-wide identification of CAMTA gene family members in rice (Oryza sativa L.) and in silico study on their versatility in respect to gene expression and promoter structure. Funct. Integr. Genomics 22, 193–214. doi: 10.1007/s10142-022-00828-w 35169940

[B21] Gao R. Luo Y. Yun F. Wu X. Wang P. Liao W. (2021). Genome-wide identification, expression profile, and alternative splicing analysis of CAMTA family genes in cucumber (Cucumis sativus L.). Agronomy 11, 1827. doi: 10.3390/agronomy11091827 30654563

[B22] Gaudelli N. M. Komor A. C. Rees H. A. Packer M. S. Badran A. H. Bryson D. I. . (2017). Programmable base editing of A• T to G• C in genomic DNA without DNA cleavage. Nature 551, 464–471. doi: 10.1038/nature24644 29160308 PMC5726555

[B23] Gómez-Porras J. L. Riaño-Pachón D. M. Dreyer I. Mayer J. E. Mueller-Roeber B. (2007). Genome-wide analysis of ABA-responsive elements ABRE and CE3 reveals divergent patterns in Arabidopsis and rice. BMC Genomics 8, 260. doi: 10.1186/1471-2164-8-260 17672917 PMC2000901

[B24] Gong P. Han J. Reddig K. Li H. S. (2007). A potential dimerization region of dCAMTA is critical for termination of fly visual response. J. Biol. Chem. 282, 21253–21258. doi: 10.1074/jbc.M701223200 17537720

[B25] Han J. Gong P. Reddig K. Mitra M. Guo P. Li H. S. (2006). The fly CAMTA transcription factor potentiates deactivation of rhodopsin, a G protein-coupled light receptor. Cell. 127, 847–858. doi: 10.1016/j.cell.2006.09.030 17110341

[B26] Hoffman N. E. (2021). Revisions to USDA biotechnology regulations: The SECURE rule. Proc. Natl. Acad. Sci. 118, e2004841118. doi: 10.1073/pnas.2004841118 34050018 PMC8179154

[B27] Hong K. Radani Y. Manda T. Chen J. Yang L. (2024). Identification and transcriptional regulation of CAMTA genes in Liriodendron chinense. Phyton. (0031-9457). 93 (3), 413–425. doi: 10.32604/phyton.2024.047739

[B28] Hou Y. Zhao L. Xie B. Hu S. Zheng Y. Jin P. (2022). EjCaM7 and EjCAMTA3 synergistically alleviate chilling-induced lignification in loquat fruit by repressing the expression of lignin biosynthesis genes. Postharvest. Biol. Technol. 192, 112010. doi: 10.1016/j.postharvbio.2022.112010 38826717

[B29] Kakar K. U. Nawaz Z. Cui Z. Cao P. Jin J. Shu Q. . (2018). Evolutionary and expression analysis of CAMTA gene family in Nicotiana tabacum yielded insights into their origin, expansion and stress responses. Sci. Rep. 8, 10322. doi: 10.1038/s41598-018-28148-9 29985386 PMC6037683

[B30] Kaplan B. Davydov O. Knight H. Galon Y. Knight M. R. Fluhr R. . (2006). Rapid transcriptome changes induced by cytosolic Ca^2+^ transients reveal ABRE-related sequences as Ca^2+^-responsive cis elements in Arabidopsis. Plant Cell 18, 2733–2748. doi: 10.1105/tpc.106.042713 16980540 PMC1626612

[B31] Kasuga M. Liu Q. Miura S. Yamaguchi-Shinozaki K. Shinozaki K. (1999). Improving plant drought, salt, and freezing tolerance by gene transfer of a single stress-inducible transcription factor. Nat. Biotechnol. 17, 287–291. doi: 10.1038/7036 10096298

[B32] Kidokoro S. Yoneda K. Takasaki H. Takahashi F. Shinozaki K. Yamaguchi-Shinozaki K. (2017). Different cold-signaling pathways function in the responses to rapid and gradual decreases in temperature. Plant Cell 29, 760–774. doi: 10.1105/tpc.16.00669 28351986 PMC5435423

[B33] Komor A. C. Kim Y. B. Packer M. S. Zuris J. A. Liu D. R. (2016). Programmable editing of a target base in genomic DNA without double-stranded DNA cleavage. Nature 533, 420–424. doi: 10.1038/nature17946 27096365 PMC4873371

[B34] Koo S. C. Choi M. S. Chun H. J. Shin D. B. Park B. S. Kim Y. H. . (2009). The calmodulin-binding transcription factor OsCBT suppresses defense responses to pathogens in rice. Mol. Cells 27, 563–570. doi: 10.1007/s10059-009-0081-4 19466605

[B35] Kumar A. Batra T. Vishwakarma H. Maurya R. Ruperao P. Yadav R. . (2024). Genome-wide analysis of the calmodulin-binding transcription activator (CAMTA) gene family in Sesamum indicum L. and its role in abiotic stress tolerance traits. Plant Stress 13, 100532. doi: 10.1016/j.stress.2024.100532 38826717

[B36] Leng X. Han J. Wang X. Zhao M. Sun X. Wang C. . (2015). Characterization of a calmodulin‐binding transcription factor from strawberry (Fragaria× ananassa). Plant Genome 8, 2014–2008. doi: 10.3835/plantgenome2014.08.0039 33228307

[B37] Li X. Huang L. Zhang Y. Ouyang Z. Hong Y. Zhang H. . (2014). Tomato SR/CAMTA transcription factors SlSR1 and SlSR3L negatively regulate disease resistance response and SlSR1L positively modulates drought stress tolerance. BMC Plant Biol. 14, 286. doi: 10.1186/s12870-014-0286-3 25348703 PMC4219024

[B38] Li D. Jin Y. Lu Q. H. Ren N. Zhang X. Y. Li L. Y. . (2025). Calmodulin1–calmodulin binding transcription activator (CAM1–CAMTA) negatively regulate the transcription of Fluoride Export Gene 1 (FEX1) to mediate fluoride transport in tea (Camellia sinensis). J. Exp. Bot. 76, 2715–2726. doi: 10.1093/jxb/eraf113 40168132 PMC12223494

[B39] Liu N. Olson E. N. (2006). Coactivator control of cardiovascular growth and remodeling. Curr. Opin. Cell Biol. 18, 715–722. doi: 10.1016/j.ceb.2006.10.003 17046230

[B40] Liu Y. Qiao Y. Liao W. (2025). Calmodulin-binding transcription factors: Roles in plant response to abiotic stresses. Plants 14, 532. doi: 10.3390/plants14040532 40006791 PMC11859506

[B41] Liu C. Tang D. (2023). Comprehensive identification and expression analysis of CAMTA gene family in Phyllostachys edulis under abiotic stress. PeerJ 11, e15358. doi: 10.7717/peerj.15358/supp-5 37180580 PMC10174056

[B42] Meer L. Mumtaz S. Labbo A. M. Khan M. J. Sadiq I. (2019). Genome-wide identification and expression analysis of calmodulin-binding transcription activator genes in banana under drought stress. Sci. Hortic. 244, 10–14. doi: 10.1016/j.scienta.2018.09.022 38826717

[B43] Miller G. Suzuki N. Ciftci-Yilmaz S. Mittler R. (2010). Reactive oxygen species homeostasis and signalling during drought and salinity stresses. Plant Cell Environ. 33, 453–467. doi: 10.1111/j.1365-3040.2009.02041.x 19712065

[B44] Morelli E. Gullà A. Amodio N. Taiana E. Neri A. Fulciniti M. . (2021). “ CRISPR interference (CRISPRi) and CRISPR activation (CRISPRa) to explore the oncogenic lncRNA network,” in Long non-coding RNAs in cancer ( Springer US, New York, NY), 189–204. doi: 10.1007/978-1-0716-1581-2_13 34160808

[B45] Müller C. W. Rey F. A. Sodeoka M. Verdine G. L. Harrison S. C. (1995). Structure of the NF-jB p50 homodimer bound to DNA. Nature 373, 311–317. doi: 10.1038/373311a0 7830764

[B46] Nayak N. Mehrotra S. Karamchandani A. N. Santelia D. Mehrotra R. (2025). Recent advances in designing synthetic plant regulatory modules. Front. Plant Sci. 16, 1567659. doi: 10.3389/fpls.2025.1567659 40241826 PMC11999978

[B47] Noman M. Jameel A. Qiang W. D. Ahmad N. Liu W. C. Wang F. W. . (2019). Overexpression of GmCAMTA12 enhanced drought tolerance in Arabidopsis and soybean. Int. J. Mol. Sci. 20, 4849. doi: 10.3390/ijms20194849 31569565 PMC6801534

[B48] Pandey N. Ranjan A. Pant P. Tripathi R. K. Ateek F. Pandey H. P. . (2013). CAMTA 1 regulates drought responses in Arabidopsis thaliana. BMC Genomics 14, 216. doi: 10.1186/1471-2164-14-216 23547968 PMC3621073

[B49] Pant P. Iqbal Z. Pandey B. K. Sawant S. V. (2018). Genome-wide comparative and evolutionary analysis of calmodulin-binding transcription activator (CAMTA) family in Gossypium species. Sci. Rep. 8, 5573. doi: 10.1038/s41598-018-23846-w 29615731 PMC5882909

[B50] Park C. Y. Lee J. H. Yoo J. H. Moon B. C. Choi M. S. Kang Y. H. . (2005). WRKY group IId transcription factors interact with calmodulin. FEBS Lett. 579, 1545–1550. doi: 10.1016/j.febslet.2005.01.057 15733871

[B51] Rahman H. Xu Y. P. Zhang X. R. Cai X. Z. (2016b). Brassica napus genome possesses extraordinary high number of CAMTA genes and CAMTA3 contributes to PAMP triggered immunity and resistance to Sclerotinia sclerotiorum. Front. Plant Sci. 7, 581. doi: 10.3389/fpls.2016.00581 27200054 PMC4854897

[B52] Rahman H. Yang J. Xu Y. P. Munyampundu J. P. Cai X. Z. (2016a). Phylogeny of plant CAMTAs and role of AtCAMTAs in nonhost resistance to Xanthomonas oryzae pv. oryzae. Front. Plant Sci. 7, 177. doi: 10.3389/fpls.2016.00177 26973658 PMC4770041

[B53] Reynolds M. Foulkes J. Furbank R. Griffiths S. King J. Murchie E. . (2012). Achieving yield gains in wheat. Plant Cell Environ. 35, 1799–1823. doi: 10.1111/j.1365-3040.2012.02588.x 22860982

[B54] Rodríguez-Leal D. Lemmon Z. H. Man J. Bartlett M. E. Lippman Z. B. (2017). Engineering quantitative trait variation for crop improvement by genome editing. Cell. 171, 470–480. doi: 10.1016/j.cell.2017.08.030 28919077

[B55] Rushton P. J. Reinstädler A. Lipka V. Lippok B. Somssich I. E. (2002). Synthetic plant promoters containing defined regulatory elements provide novel insights into pathogen-and wound-induced signaling. Plant Cell 14, 749–762. doi: 10.1105/tpc.010412 11971132 PMC150679

[B56] Saeidi K. Zare N. Baghizadeh A. Asghari-Zakaria R. (2019). Phaseolus vulgaris genome possesses CAMTA genes, and phavuCAMTA1 contributes to the drought tolerance. J. Genet. 98, 31. doi: 10.1007/s12041-019-1069-2 30945676

[B57] Saidi A. Hajibarat Z. (2021). Evolutionary and expression analysis of CAMTA gene family in three species (Arabidopsis, Maize and Tomato), and gene expression in response to developmental stages. Research Square [Preprint]. doi: 10.21203/rs.3.rs-678620/v1

[B58] Schulman A. H. Hartung F. Smulders M. J. Sundström J. F. Wilhelm R. Rognli O. A. . (2025). Proposed EU NGT legislation in light of plant genetic variation. Plant Biotechnol. J. 23, 4261–4270. doi: 10.1111/pbi.70228 40586315 PMC12483989

[B59] Schwartz R. J. Schneider M. D. (2006). CAMTA in cardiac hypertrophy. Cell. 125, 427–429. doi: 10.1016/j.cell.2006.04.015 16678087

[B60] Shen C. Yang Y. Du L. Wang H. (2015). Calmodulin-binding transcription activators and perspectives for applications in biotechnology. Appl. Microbiol. Biotechnol. 99, 10379–10385. doi: 10.1007/s00253-015-6966-6 26450508

[B61] Song K. Backs J. McAnally J. Qi X. Gerard R. D. Richardson J. A. . (2006). The transcriptional coactivator CAMTA2 stimulates cardiac growth by opposing class II histone deacetylases. Cell. 125, 453–466. doi: 10.1016/j.cell.2006.02.048 16678093

[B62] Song B. Yu J. Li X. Li J. Fan J. Liu H. . (2024). Increased DNA methylation contributes to the early ripening of pear fruits during domestication and improvement. Genome Biol. 25, 87. doi: 10.1186/s13059-024-03220-y 38581061 PMC10996114

[B63] Sonkar K. Kamali S. Kumar A. Deepika D. Ankit A. Singh A. (2025). Genomic, structural, and molecular analysis of calmodulin-binding transcriptional activators (CAMTAs) suggests their role in plant development and abiotic stress tolerance in chickpea. Comput. Struct. Biotechnol. J. 27, 3824–3383. doi: 10.1016/j.csbj.2025.08.032 40994538 PMC12455000

[B64] Szymczyk P. Majewska M. (2024). Plant synthetic promoters. Appl. Sci. 14, 4877. doi: 10.3390/app14114877 30654563

[B65] Tokizawa M. Kobayashi Y. Saito T. Kobayashi M. Iuchi S. Nomoto M. . (2015). Sensitive to proton rhizotoxicity1, calmodulin binding transcription activator2, and other transcription factors are involved in aluminum-activated malate transporter1 expression. Plant Physiol. 167, 991–1003. doi: 10.1104/pp.114.256552 25627216 PMC4348791

[B66] USDA APHIS . (2021). Sustainable, Ecological, Consistent, Uniform, Responsible, Efficient (SECURE) Rule. United States Department of Agriculture, Animal and Plant Health Inspection Service. Available online at: https://www.aphis.usda.gov/biotechnology/regulations/secure-rule (Accessed January 16, 2026).

[B67] Vuong-Brender T. T. Flynn S. Vallis Y. Sönmez S. E. de Bono M. (2021). Neuronal calmodulin levels are controlled by CAMTA transcription factors. Elife 10, e68238. doi: 10.7554/eLife.68238 34499028 PMC8428840

[B68] Wang G. Zeng H. Hu X. Zhu Y. Chen Y. Shen C. . (2015). Identification and expression analyses of calmodulin-binding transcription activator genes in soybean. Plant Soil 386, 205–221. doi: 10.1007/s11104-014-2267-6 30311153

[B69] Wankerl L . (2016). Characterization of CAMTA1 and Nkx2.2 in the context of glioblastoma cancer stem cell biology. [dissertation]. Regensburg, Germany: Universität Regensburg. doi: 10.5283/epub.33258

[B70] Wei M. Xu X. Li C. (2017). Identification and expression of CAMTA genes in Populus trichocarpa under biotic and abiotic stress. Sci. Rep. 7, 17910. doi: 10.1038/s41598-017-18219-8 29263356 PMC5738416

[B71] Xiao P. Feng J. W. Zhu X. T. Gao J. (2021). Evolution analyses of CAMTA transcription factor in plants and its enhancing effect on cold-tolerance. Front. Plant Sci. 12, 758187. doi: 10.3389/fpls.2021.758187 34790215 PMC8591267

[B72] Xu L. Liu Y. (2024). Identification, design, and application of noncoding cis-regulatory elements. Biomolecules 14, 945. doi: 10.3390/biom14080945 39199333 PMC11352686

[B73] Yang F. Dong F. S. Hu F. H. Liu Y. W. Chai J. F. Zhao H. . (2020a). Genome-wide identification and expression analysis of the calmodulin-binding transcription activator (CAMTA) gene family in wheat (Triticum aestivum L.). BMC Genet. 21, 105. doi: 10.1186/s12863-020-00916-5 32928120 PMC7491182

[B74] Yang X. Jia Z. C. Liu Y. Wang X. Chen J. J. Liu Y. G. . (2026). From shared mechanisms to precision breeding: engineering cold and drought cross-tolerance in crops. Int. J. Mol. Sci. 27, 2497. doi: 10.3390/ijms27052497 41828711 PMC12986518

[B75] Yang C. Li Z. Cao X. Duan W. Wei C. Zhang C. . (2022a). Genome-wide analysis of calmodulin binding transcription activator (CAMTA) gene family in peach (Prunus persica L. Batsch) and ectopic expression of PpCAMTA1 in Arabidopsis camta2, 3 mutant restore plant development. Int. J. Mol. Sci. 23, 10500. doi: 10.3390/ijms231810500 36142414 PMC9499639

[B76] Yang Y. Li J. Yao M. Chen S. (2024). Genome-Wide Identification of CAMTA Gene Family in Oat (Avena sativa) and Expression Analysis under Various Abiotic Stresses. Agronomy 14, 2053. doi: 10.3390/agronomy14092053 30654563

[B77] Yang T. Peng H. Whitaker B. D. Conway W. S. (2012). Characterization of a calcium/calmodulin-regulated SR/CAMTA gene family during tomato fruit development and ripening. BMC Plant Biol. 12, 1–13. doi: 10.1186/1471-2229-12-19 22330838 PMC3292969

[B78] Yang T. Poovaiah B. W. (2002). A calmodulin-binding/CGCG box DNA-binding protein family involved in multiple signaling pathways in plants. J. Biol. Chem. 277, 45049–45058. doi: 10.1074/jbc.M207941200 12218065

[B79] Yang H. Ren S. Yu S. Pan H. Li T. Ge S. . (2020b). Methods favoring homology-directed repair choice in response to CRISPR/Cas9 induced-double strand breaks. Int. J. Mol. Sci. 21, 6461. doi: 10.3390/ijms21186461 32899704 PMC7555059

[B80] Yang L. Zhao Y. Zhang G. Shang L. Wang Q. Hong S. . (2022b). Identification of CAMTA gene family in Heimia myrtifolia and expression analysis under drought stress. Plants 11, 3031. doi: 10.3390/plants11223031 36432758 PMC9698416

[B81] You W. Zhang J. Ru X. Xu F. Wu Z. Jin P. . (2024). CaCl_2_ promoted phenolics accumulation via the CmCAMTA4-mediated transcriptional activation of phenylpropane pathway and energy metabolism in fresh-cut cantaloupe. Postharvest. Biol. Technol. 207, 112599. doi: 10.1016/j.postharvbio.2023.112599 38826717

[B82] Yu J. Song B. Gu K. Cao B. Zhao K. Wu J. . (2023). Genome-Wide identification and expression analysis of CAMTA gene family implies PbrCAMTA2 involved in fruit softening in Pear. Horticulturae 9, 467. doi: 10.3390/horticulturae9040467 30654563

[B83] Yuan J. Shen C. Chen B. Shen A. Li X. (2021). Genome-wide characterization and expression analysis of CAMTA gene family under salt stress in Cucurbita moschata and Cucurbita maxima. Front. Genet. 12, 647339. doi: 10.3389/fgene.2021.647339 34220934 PMC8249228

[B84] Yue R. Lu C. Sun T. Peng T. Han X. Qi J. . (2015). Identification and expression profiling analysis of calmodulin-binding transcription activator genes in maize (Zea mays L.) under abiotic and biotic stresses. Front. Plant Sci. 6, 576. doi: 10.3389/fpls.2015.00576 26284092 PMC4516887

[B85] Zaman S. Hassan S. S. U. Ding Z. (2022). The role of calmodulin binding transcription activator in plants under different stressors: Physiological, biochemical, molecular mechanisms of Camellia sinensis and its current progress of CAMTAs. Bioengineering 9, 759. doi: 10.3390/bioengineering9120759 36550965 PMC9774361

[B86] Zhang J. Li Y. Zuo X. Ru X. You W. Xu F. . (2023). The interaction between DcCaM7 and DcCAMTA6 promotes γ-aminobutyric acid (GABA) accumulation via regulating GABA shunt in fresh-cut carrots. Postharvest. Biol. Technol. 204, 112478. doi: 10.1016/j.postharvbio.2023.112478 38826717

[B87] Zhang J. Pan X. Ge T. Yi S. Lv Q. Zheng Y. . (2019). Genome-wide identification of citrus CAMTA genes and their expression analysis under stress and hormone treatments. J. Hortic. Sci. Biotechnol. 94, 331–340. doi: 10.1080/14620316.2018.1504631 37339054

[B88] Zheng K. Li M. Yang Z. He C. Wu Z. Tong Z. . (2024). The vital role of the CAMTA gene family in phoebe bournei in response to drought, heat, and light stress. Int. J. Mol. Sci. 25, 9767. doi: 10.3390/ijms25189767 39337256 PMC11432206

[B89] Zhou L. Ullah F. Zou J. Zeng X. (2025). Molecular and physiological responses of plants that enhance cold tolerance. Int. J. Mol. Sci. 26, 1157. doi: 10.3390/ijms26031157 39940925 PMC11818088

[B90] Zhou Q. Zhao M. Xing F. Mao G. Wang Y. Dai Y. . (2022). Identification and expression analysis of CAMTA genes in tea plant reveal their complex regulatory role in stress responses. Front. Plant Sci. 13, 910768. doi: 10.3389/fpls.2022.910768 35712571 PMC9196129

[B91] Zhu H. Li C. Gao C. (2020). Applications of CRISPR–Cas in agriculture and plant biotechnology. Nat. Rev. Mol. Cell Biol. 21, 661–677. doi: 10.1038/s41580-020-00288-9 32973356

[B92] Zhu X. Wang P. Bai Z. Herde M. Ma Y. Li N. . (2022b). Calmodulin‐like protein CML24 interacts with CAMTA2 and WRKY46 to regulate ALMT1‐dependent Al resistance in Arabidopsis thaliana. New Phytol. 233, 2471–2487. doi: 10.1111/nph.17812 34665465

[B93] Zhu X. Wang B. Wei X. Du X. (2022a). Characterization of the CqCAMTA gene family reveals the role of CqCAMTA03 in drought tolerance. BMC Plant Biol. 22, 428. doi: 10.1186/s12870-022-03817-0 36071408 PMC9450354

[B94] Zou C. Sun K. Mackaluso J. D. Seddon A. E. Jin R. Thomashow M. F. . (2011). Cis-regulatory code of stress-responsive transcription in Arabidopsis thaliana. Proc. Natl. Acad. Sci. 108, 14992–14997. doi: 10.1073/pnas.1103202108 21849619 PMC3169165

[B95] Zsögön A. Čermák T. Naves E. R. Notini M. M. Edel K. H. Weinl S. . (2018). De novo domestication of wild tomato using genome editing. Nat. Biotechnol. 36, 1211–1216. doi: 10.1038/nbt.4272 30272678

